# Supporting Implementation of the National Standards for Cancer Survivorship Care: Development of the Cancer Survivorship Maturity Model (CSMM)

**DOI:** 10.21203/rs.3.rs-10021745/v1

**Published:** 2026-06-28

**Authors:** Heidi Dowst, Maryem Shirzadi, Martha Mims, Susan Hilsenbeck, Liwei Wang, Hoda Badr

**Affiliations:** Baylor College of Medicine; Baylor College of Medicine; Baylor College of Medicine; Baylor College of Medicine; The University of Texas at Austin; Sidney Kimmel Comprehensive Cancer Center

**Keywords:** Cancer survivorship, Survivorship care standards, Organizational readiness, Maturity model

## Abstract

**Purpose.:**

National survivorship care standards define the domains of care that healthcare organizations should deliver, yet few tools exist to assess readiness for implementation. We developed and preliminarily evaluated the Cancer Survivorship Maturity Model (CSMM), a framework designed to assess organizational readiness to implement survivorship care standards.

**Methods.:**

We conducted a mixed-methods, participatory model development study. A preliminary CSMM, grounded in national survivorship care standards, was refined through five multidisciplinary focus groups at a comprehensive cancer center using consensus-building techniques adapted from Nominal Group Technique. Participants completed prioritization exercises, contributed to iterative model refinement, and assessed the maturity of survivorship care delivery in their practice settings. Qualitative analysis identified model refinement needs and implementation barriers. The final model was distributed to external stakeholders from the National Cancer Institute Survivorship Supplement Awardees network for preliminary evaluation.

**Results.:**

Fourteen internal participants and 12 external respondents contributed to model development and evaluation. The final CSMM included seven domains across five maturity levels ranging from Ad Hoc to Optimized. Participants identified differences in prioritization across clinical impact, implementation feasibility, and perceived patient importance. Across internal and external applications, survivorship care maturity was generally low, with most domains clustering between Levels 1 and 2. Qualitative findings identified barriers, including unclear workflows, limited resources, insufficient time, and technical constraints.

**Conclusions.:**

The CSMM provides a structured approach to assess organizational readiness for survivorship care implementation and to identify domain-specific gaps.

**Implications for Cancer Survivors.:**

By helping institutions target areas for improvement and resource investment, the CSMM may support more consistent, coordinated, and patient-centered survivorship care delivery.

## Background

A cancer diagnosis has far-reaching effects that extend well beyond the direct treatment of disease, influencing nearly every aspect of a patient’s physical, emotional, social, and financial well-being. The National Cancer Institute (NCI) defines survivorship as beginning at the time of diagnosis and continuing throughout the balance of life, reflecting the need for coordinated care across the cancer continuum [1]. Survivors frequently experience persistent symptom burden, financial hardship, disruptions in social and occupational functioning, and ongoing needs for health promotion, supportive care, and communication across oncology and primary care settings [1–4]. As the number of cancer survivors continues to grow, healthcare systems are increasingly expected to deliver survivorship care that is comprehensive, coordinated, and responsive to these multidimensional needs.

In response to this need, national organizations including the Institute of Medicine, the NCI, and the American Society of Clinical Oncology have called for survivorship care to become a routine component of high-quality cancer care [1–3]. More recently, the National Standards for Cancer Survivorship Care have provided a more explicit framework for the domains of care that healthcare organizations should be prepared to address, including surveillance, symptom management, supportive services, care coordination, and patient-centered follow-up [1]. These standards represent an important advance for the field because they move survivorship care from a broadly endorsed principle to a more actionable organizational expectation. However, standards alone do not ensure implementation. Healthcare organizations vary substantially in the infrastructure, workflows, staffing models, clinical integration, and data systems needed to operationalize survivorship care consistently across practice settings [5].

A major challenge for the field, therefore, is not only defining what survivorship care should include, but also determining whether organizations are prepared to implement it. Although survivorship frameworks and guidelines describe recommended domains of care, fewer tools exist to assess an institution’s operational readiness to deliver those domains in routine practice.

Several established survivorship quality frameworks and accreditation standards address elements of survivorship care delivery, including Commission on Cancer Standard 4.8 monitoring metrics, the LIVESTRONG Cancer Survivorship Center of Excellence Network indicators, ASCO Quality Oncology Practice Initiative (QOPI) survivorship measures, and National Accreditation Program for Breast Centers (NAPBC) standards [6–9]. These efforts have advanced the field by defining core survivorship services and establishing performance expectations; however, they primarily function as checklists or compliance-oriented metrics and do not provide a structured, quantitative framework for assessing an organization’s stage of implementation across domains. In contrast, maturity models are widely used in other areas of healthcare and informatics and are designed to characterize progressive levels of organizational capability and readiness [10, 11]. While originally developed to evaluate process capability and organizational performance, maturity models have increasingly been applied in healthcare and biomedical informatics to assess the readiness of systems, workflows, and data-enabled practices for implementation and scale [12, 13]. Their value lies not only in identifying strengths and gaps, but also in providing a practical roadmap for staged improvement. In the context of cancer survivorship, a maturity model may offer a way to translate national standards into an actionable organizational assessment framework that can support both local implementation planning and broader cross-institutional learning.

To address these practical implementation needs, this study aimed to develop and preliminarily evaluate the Cancer Survivorship Maturity Model (CSMM), a clinician-informed framework grounded in the National Standards for Cancer Survivorship Care and designed to assess organizational readiness to implement survivorship care in real-world oncology settings. The CSMM was intended to characterize institutional capacity across key survivorship domains and to define progressive levels of implementation maturity that could guide quality improvement and infrastructure development. At the institutional level, such a model may help organizations identify specific readiness gaps and prioritize survivorship care investments. At a broader level, it may help illuminate common implementation barriers and variation in survivorship care readiness across settings, with implications for dissemination, technical assistance, and future survivorship research. To our knowledge, such maturity-based frameworks have been minimally applied in cancer survivorship, representing a key gap that the CSMM was designed to address.

## Methods

### Study Design

We conducted a mixed-methods, participatory model development study to develop and preliminarily evaluate the CSMM, an organizational readiness framework designed to assess implementation of survivorship care standards in clinical oncology settings. Model development was informed by structured focus groups using consensus-building techniques adapted from the Nominal Group Technique (NGT), followed by external stakeholder evaluation for transferability [14–16]. This design was selected to ensure that the model was both conceptually grounded in national survivorship care standards and informed by real-world clinical workflows and implementation contexts. NGT-informed approaches are commonly used in health services research to elicit expert perspectives, reduce dominance by individual participants, and support structured prioritization and refinement of emerging frameworks [17, 18].

### Setting and Participants

#### Internal focus groups

Five focus groups were conducted at the Dan L Duncan Comprehensive Cancer Center. Participants were recruited using purposive sampling to capture a range of perspectives across disciplines involved in survivorship care delivery. Eligible participants included multidisciplinary clinicians and supportive care professionals with direct experience in survivorship care pathways, including oncology, primary care, nursing, social work, rehabilitation, and psychosocial services. This approach was used to ensure that the model reflected the interdisciplinary nature of survivorship care implementation. We aimed to enroll 4 to 6 participants per group, consistent with recommended group sizes for focus group and adapted NGT studies [17, 18]. Discussions were moderated by a researcher with expertise in maturity model development [HD] and a second researcher with domain expertise in cancer survivorship care [HB].

##### External stakeholder evaluation

Following internal model refinement, the CSMM was distributed electronically to stakeholders affiliated with the National Cancer Institute Survivorship Supplement Awardees (NCISS) network to obtain preliminary feedback on usability and applicability across institutions. External participants were invited to complete the model as an institutional self-assessment of readiness to implement survivorship care standards.

##### Development of the Preliminary CSMM

A preliminary (“strawman”) version of the CSMM was developed prior to focus group convening using the National Guidelines for Cancer Survivorship Care as the foundational framework [1]. The goal was to translate these standards into an organizational readiness instrument suitable for structured maturity assessment. Because survivorship care standards define priority domains of care rather than discrete organizational capabilities, direct one-to-one translation into a maturity model was not methodologically optimal. Maturity models require domains that are conceptually distinct, operationally assessable, and interpretable by respondents in relation to existing workflows, infrastructure, and team roles [10–13, 19, 20]. Accordingly, conceptually related survivorship care elements that shared common implementation pathways, staffing dependencies, and referral mechanisms were consolidated into broader operational domains. This approach was intended to reduce redundancy, improve interpretability, and enhance feasibility of use while preserving alignment with the underlying survivorship care standards.

The preliminary CSMM included seven categories that map to National Guidelines for Cancer Survivorship Care domains (Supplemental Table 1): Survivorship Care Planning, Risk of Recurrence, Specialty Care, Financial Hardship/Toxicity, Lifestyle Behaviors & Supportive Services, Practical & Social Impacts, Physical & Psychological Effects. For example, supportive services that are often delivered through shared referral and navigation workflows, including nutrition, exercise, and psychosocial support, were grouped into a single category. Similarly, practical and social concerns were grouped to reflect shared screening and referral pathways in routine care. This consolidation was designed to align the model with how survivorship care is operationalized in practice, while maintaining conceptual consistency with the NCI standards.

Each category was represented across five maturity levels, adapted from established maturity model frameworks [10–13, 19, 20], ranging from Level 1 (Ad Hoc) to Level 5 (Optimized). Intermediate levels were labeled Planned, Unified, and Reportable to reflect increasing degrees of standardization, integration, and organizational capability. Draft descriptors for each maturity level were developed to reflect observable institutional characteristics, including workflows, staffing, referral processes, documentation practices, and use of data to support survivorship care delivery. The preliminary model is provided in Supplemental Table 1.

##### Focus Group Procedures

Each focus group followed a structured process designed to refine model content, assess perceived implementation priorities, and identify organizational barriers relevant to survivorship care readiness. Participants first completed a brief demographic survey capturing clinical role and practice context. The moderator then introduced the study objectives and provided a standardized overview of survivorship care standards and maturity model concepts. Participants completed an initial prioritization exercise using dot voting, a low-burden adaptation of NGT multi-voting [17, 18]. They were asked to rank CSMM domains based on three implementation-oriented questions: (1) which domain would have the greatest clinical impact if effectively implemented; (2) which domain would be easiest to implement; and (3) which domain patients would prioritize most highly. This exercise was designed to capture differences between clinical importance, implementation feasibility, and perceived patient-centered priorities.

Following this exercise, the moderator reviewed the structure of the strawman model and the meaning of the five maturity levels to ensure a shared understanding of the framework. Participants then reviewed assigned domains and proposed modifications to the draft maturity level descriptors. The goal of this step was to enhance clarity, face validity, and real-world relevance by ensuring that each level reflected recognizable institutional capabilities and workflows.

Participants reconvened for facilitated group discussion of proposed modifications. Discussions focused on refining domain definitions, resolving ambiguities, and identifying cross-cutting implementation challenges. Focus group discussions were audio recorded, and moderator notes were documented to support model refinement and qualitative analysis. These materials were used to iteratively revise the model. Focus groups were conducted using a pragmatic stopping approach rather than a formal saturation criterion, consistent with prior work highlighting the ambiguity in defining and operationalizing saturation and the importance of aligning sample size decisions with study purpose and analytic approach [21].

Participants then completed a current-state assessment by rating the perceived maturity of their own practice setting for each domain. Aggregate responses were displayed in real time using radar plots to visually summarize domain-level maturity patterns. These visualizations were used as a structured reflection prompt, allowing participants to comment on areas of relative strength and weakness and to discuss whether institutional maturity aligned with domains they had previously identified as high priority, clinically impactful, or most relevant to patients [22]. This facilitated interpretation of both quantitative ratings and qualitative perceptions of implementation gaps.

#### External Validation

While no established maturity model evaluation framework has been previously published in the literature, external validation of developed maturity models is identified as necessary to ensure application in other healthcare settings and assure that limited bias is incorporated into the models [12]. Following refinement through internal focus groups, the CSMM was implemented in REDCap and distributed to external stakeholders within the NCISS network [23, 24].

Respondents completed the model as an institutional self-assessment of readiness to implement survivorship care standards.

To enhance interpretability, respondents were shown their individual domain scores alongside aggregated responses from prior participants. External evaluation was intended to provide an initial assessment of the model’s usability, transferability, and ability to capture variation in survivorship care readiness across institutional contexts.

##### Analysis

This study used a mixed-methods analytic approach to integrate structured prioritization data, maturity ratings, and qualitative focus group feedback.

##### Quantitative Analysis

Prioritization responses from the focus groups were summarized using weighted rankings. For each prioritization question, first-, second-, and third-place rankings were assigned 3, 2, and 1 points, respectively, and summed across participants to generate domain-level scores. Maturity ratings were summarized descriptively by domain and participant group. Means and standard deviations were calculated for internal and external respondents. Visualizations, including radar plots, were used to summarize domain-level maturity patterns and support interpretation of variation within and between respondent groups. To explore differences in maturity ratings between internal and external respondents, ordinal logistic regression models were conducted for each domain. These analyses were exploratory and intended to characterize patterns of variation rather than to test predefined hypotheses.

##### Qualitative Analysis

Audio recordings, moderator notes, and documented model modifications from the focus groups were reviewed by the research team using an applied qualitative approach. Analysis focused on two prespecified areas: (1) feedback that informed revision of CSMM domains and maturity level descriptors, and (2) recurring implementation barriers related to survivorship care readiness. Through iterative review and discussion, the research team identified recurrent patterns and representative quotations that informed refinement of the model and interpretation of quantitative findings.

##### Integration of mixed-methods findings

Quantitative and qualitative findings were integrated during interpretation to assess whether domain prioritization, maturity ratings, and focus group reflections converged or diverged across survivorship care domains. This integration was used to strengthen interpretation of the CSMM’s usability and the organizational barriers affecting survivorship care implementation.

## Results

### Participant Characteristics

A total of 14 multidisciplinary participants contributed to model development across five internal focus groups. Participants represented a range of disciplines involved in survivorship care delivery, including oncology, primary care, nursing, social work, rehabilitation, and psychosocial services, and practiced across diverse care settings within the cancer center, including academic, safety-net, and Veterans Affairs environments. In addition, 12 external respondents from the NCISS network participated in preliminary external evaluation. Detailed participant characteristics are presented in Table 1.

### Prioritization of Survivorship Domains

**Figure 1:**
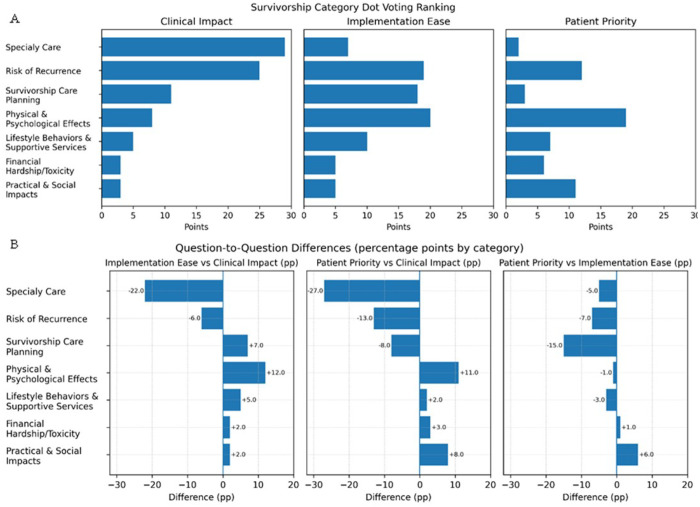
Focus group prioritization of cancer survivorship care categories across clinical impact, implementation ease, and patient priority. **(A)** Focus group rankings of cancer survivorship categories ordered by perceived clinical impact, with corresponding rankings shown for implementation ease and patient priority. **(B)**Divergent bar chart displaying absolute percentage-point differences in rankings between Question 1 (clinical impact), Question 2 (implementation ease), and Question 3 (patient priority), highlighting areas of concordance and divergence across prioritization dimensions. CSMM categories were mapped to the National Standards for Cancer Survivorship Care Guideline domains as shown in Footnote a.

Participants ranked survivorship domains based on perceived clinical impact, ease of implementation, and perceived patient priority (Fig. 1A). Risk of Recurrence, Specialty Care, and Survivorship Care Planning were perceived as the domains with the greatest potential clinical impact. Specialty Care, Risk of Recurrence, and Survivorship Care Planning were also perceived as the easiest domains to implement. In contrast, Physical & Psychological Effects, Risk of Recurrence, and Financial Hardship/Toxicity were more often identified as the highest-priority domains from the patient perspective. Figure 1. Focus group prioritization of cancer survivorship care categories across clinical impact, implementation ease, and patient priority. **(A)** Focus group rankings of cancer survivorship categories ordered by perceived clinical impact, with corresponding rankings shown for implementation ease and patient priority. **(B)** Divergent bar chart displaying absolute percentage-point differences in rankings between Question 1 (clinical impact), Question 2 (implementation ease), and Question 3 (patient priority), highlighting areas of concordance and divergence across prioritization dimensions. CSMM categories were mapped to the National Standards for Cancer Survivorship Care Guideline domains as shown in Footnote a.

Comparisons across ranking dimensions demonstrated only modest alignment, with notable divergence across domains (Fig. 1B). In particular, Specialty Care was rated highly for clinical impact but less highly for perceived patient priority, whereas domains related to symptom burden and financial hardship were more strongly associated with patient-centered priorities. This pattern was supported by low overall concordance across rankings (Kendall’s W = 0.056) and modest pairwise rank correlations. Clinical Impact and Implementation Ease demonstrated moderate positive alignment (Spearman’s rho = 0.527), as did Implementation Ease and perceived Patient Priority (rho = 0.505). In contrast, Clinical Impact and perceived Patient Priority showed weak negative alignment (rho = − 0.252), suggesting that domains perceived as most influential for clinical outcomes were not always those perceived as most important to patients.

### Qualitative Refinement of the Cancer Survivorship Maturity Model

Focus group discussions informed iterative refinement of the CSMM, resulting in clarification of domain definitions and maturation descriptors. Supplemental Table 1 presents the initial strawman model, Supplemental Table 2 summarizes focus group modifications, and Supplemental Table 3 reflects the final revised CSMM used for external evaluation. Participants generally endorsed the overall seven-domain structure but identified areas of conceptual overlap and ambiguity that required refinement. In particular, participants noted that practical and social challenges often intersect with financial hardship, reflecting shared underlying drivers such as employment disruption and treatment-related costs. While participants did not recommend combining these domains, they emphasized the need for clearer operational distinctions to support accurate assessment. As one participant noted, “practical and social issues bleed into financial issues,” underscoring the importance of distinguishing these domains while acknowledging their interrelated nature. Similarly, participants discussed whether psychological effects should be separated from physical symptom management, citing differences in clinical ownership and referral pathways. Ultimately, participants supported maintaining integrated domains with clearer descriptors that reflected how these issues are addressed in practice.

Across focus groups, participants emphasized that maturity level descriptions should reflect observable institutional processes, including role delineation, referral workflows, and availability of resources. Revisions focused on improving clarity, reducing ambiguity, and ensuring that each level corresponded to recognizable organizational capabilities. Later focus groups reported minimal additional modifications, suggesting stabilization of the model indicating a pragmatic stopping point. For example, one participant in the final focus group remarked, “I don’t have many changes to suggest; it looks pretty good,” reflecting stabilization of the model.

### Internal Current-State Maturity Assessment

**Figure 2:**
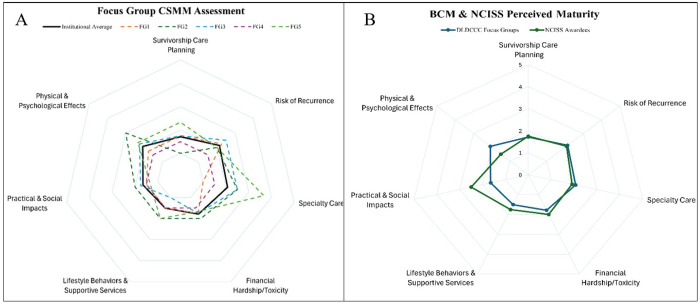
CSMM Assessment Visualizations. **(A)** Radar plot displaying current-state institutional maturity ratings across cancer survivorship categories for BCM focus groups. Individual focus group responses are shown as colored dashed lines, and the mean BCM focus group rating is displayed as a solid black line. **(B)**Mean CSMM category ratings for external NCISS respondents (green; *n* = 12) and BCM focus groups (blue; *n* = 14). Physical Symptom Management received the lowest mean rating (1.5), while Practical and Social Support received the highest mean rating (2.5).

Participants applied the CSMM to assess the maturity of survivorship care delivery within their primary practice settings (Table 2; Fig. 2A). Across domains, maturity ratings were generally low to moderate, with most domains clustering between Levels 1 (Ad Hoc) and 2 (Planned), indicating limited standardization and integration of survivorship care processes. Among internal respondents, Risk of Recurrence and Specialty Care demonstrated relatively higher average maturity compared with other domains, whereas Lifestyle Behaviors & Supportive Services and Practical & Social Impacts were among the lowest-rated areas. These findings suggest that surveillance and referral processes may be more established, while broader supportive care services remain less systematically implemented.

Variation in maturity ratings across participants was observed, particularly in domains such as Specialty Care, which ranged from a mean of 1.00 in one focus group to 3.67 in another. This variability was also reflected in the relatively larger standard deviation for Specialty Care compared with other domains (Table 3), suggesting meaningful differences in survivorship care infrastructure across clinical settings. Radar plot visualization further demonstrated variability across domains and settings, highlighting uneven development of survivorship care capabilities within the institution (Fig. 2A).

#### External Preliminary Evaluation

External respondents from the NCISS network completed the CSMM as an institutional self-assessment of survivorship care readiness (Table 3; Fig. 2B). Overall, patterns of domain-level maturity were similar between internal and external groups, with mean scores in both groups remaining in the lower range of the maturity scale. The overall mean maturity rating was 1.85 among internal respondents and 1.93 among external respondents, representing a difference of only 0.08 points on the 5-point scale.

**Figure 3:**
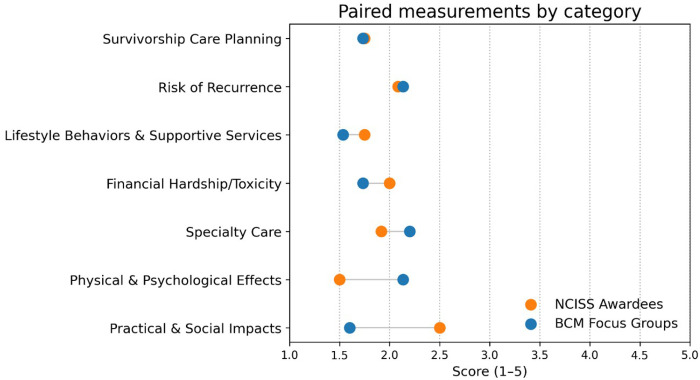
Visualization of mean score differences between BCM and NCISS respondents across cancer survivorship care categories. Survivorship Care Planning and Risk of Recurrence show the least between-site variation, whereas Practical & Social Impacts and Physical & Psychological Effects demonstrate the greatest between-site differences. Across all categories, between-site variation is small relative to the full evaluation scale.

Across domains, Practical & Social Impacts was rated relatively higher among external respondents, whereas Physical & Psychological Effects and Lifestyle Behaviors & Supportive Services remained among the lowest-rated domains. Comparative visualization demonstrated close alignment between internal and external respondents in most domains, with only modest differences observed (Fig. 3).

Social Impacts and Physical & Psychological Effects demonstrate the greatest between-site differences. Across all categories, between-site variation is small relative to the full evaluation scale.

Exploratory ordinal logistic regression analyses indicated limited evidence of systematic differences between internal and external groups across domains (Table 4). Although Practical & Social Impacts showed a statistically significant difference between internal and external respondents, interpretation is limited by small sample sizes and wide confidence intervals. Overall, these findings suggest that the CSMM is interpretable and applicable across institutional contexts and may capture common patterns in survivorship care readiness.

#### Cross-Cutting Implementation Themes

Qualitative analysis of focus group discussions identified several recurring barriers to survivorship care implementation (Table 5). These included limited availability of institutional resources, insufficient clinician time, lack of technical infrastructure, unclear processes and role delineation, and variation in survivorship care needs across cancer types and clinical settings. Among these, lack of clearly defined processes and responsibilities was the most frequently cited barrier (5 mentions), followed by clinician time constraints (4), variation in care across cancer types and settings (3), limited resources (3), and technical barriers (2). Cost or funding constraints were also noted, although less frequently (1). Participants described challenges related to fragmented workflows, lack of ownership of survivorship care tasks, and limited integration of electronic health record systems across care settings. As one participant noted, “there is a lack of workflow clarity about the process and roles,” highlighting the importance of clearly defined responsibilities in survivorship care delivery. Participants also emphasized that survivorship care is resource-intensive and often not supported by dedicated funding or protected time, limiting the ability of clinicians to implement recommended care components.

## Discussion

In this mixed-methods study, we developed and preliminarily evaluated a Cancer Survivorship Maturity Model (CSMM) to assess organizational readiness to implement survivorship care standards. Across internal and external respondents, survivorship care processes were generally rated at early stages of maturity, with most domains clustering between ad hoc and planned levels of implementation. Participants also identified meaningful differences in how survivorship domains were prioritized depending on whether they were viewed through a lens of clinical impact, implementation feasibility, or perceived patient importance. Qualitative findings further highlighted recurring organizational barriers, including unclear workflows, limited resources, insufficient time, and fragmented systems. Taken together, these findings suggest that while survivorship care is widely recognized as a priority, healthcare organizations may lack the infrastructure required to implement survivorship care standards consistently.

One of the most informative findings in this study was the divergence observed across domain prioritization. Domains perceived by respondents as having the greatest potential clinical impact were not always those perceived as easiest to implement or most important to patients. This distinction is important because survivorship care implementation often requires institutions to make practical decisions about where to invest limited resources. Our findings suggest that survivorship program development may benefit from considering multiple implementation lenses simultaneously, including expected impact on clinical outcomes, operational feasibility, and responsiveness to survivor priorities. In this sense, the prioritization exercise may have value beyond model development by offering institutions a structured approach to survivorship care planning and resource allocation.

Across both internal and external respondents, maturity ratings were tightly clustered around the lower end of the five-level scale, with overall mean ratings approximating Level 2. This pattern suggests that represented organizations may have foundational survivorship care elements in place, such as individual clinician practices, isolated referral pathways, or basic documentation supports, but may not yet have standardized, integrated, and reportable survivorship workflows. This interpretation is particularly important in the context of the National Cancer Institute’s Standards for Survivorship Care, which articulates a comprehensive vision for survivorship delivery but may exceed the operational capacity of many institutions in their current state. Assessment and screening for Risk of Recurrence was the most mature domain, indicating that institutions remain more capable of delivering clinically embedded surveillance activities than implementing comprehensive psychosocial, lifestyle behavioral, and practical survivorship services.

These findings highlight a key implementation gap in cancer survivorship care: national standards define what survivorship care should include, but less guidance exists regarding how healthcare organizations can assess their readiness to deliver it. The CSMM was designed to address this gap by translating survivorship care standards into an operational readiness framework that institutions can use to identify strengths, gaps, and staged opportunities for improvement. Rather than functioning as a checklist of services, the model is intended to help organizations understand whether they have the workflows, staffing, referral pathways, documentation practices, and infrastructure needed to implement survivorship care reliably. In this way, the CSMM may be useful not only for internal quality improvement, but also for informing institutional planning, implementation strategy, and potentially future survivorship-focused funding and technical assistance efforts. Importantly, by distinguishing between domains at earlier versus more advanced stages of maturity, the CSMM may also help institutions prioritize where to direct limited resources, implementation effort, and infrastructure investment in order to strengthen survivorship care delivery over time.

The qualitative findings provide additional insight into how institutions might use the model in practice. Participants frequently described implementation barriers that were organizational rather than conceptual, including unclear ownership of survivorship tasks, lack of technical integration, and insufficient time to address survivorship needs during clinical encounters. Importantly, participants also identified practical opportunities for improvement, such as embedding patient-reported needs screening into clinic workflows and creating more reliable referral triggers for supportive services. These examples suggest that survivorship care readiness may be improved not only through additional resources, but also through better workflow design, role clarity, and systems integration. As such, the CSMM may be especially useful as a planning tool for identifying where survivorship care processes break down and where implementation supports are most needed.

This study has several strengths. First, the CSMM was developed using a participatory, mixed-methods process that incorporated multidisciplinary perspectives from clinicians and supportive care professionals directly involved in survivorship care delivery. Second, the model was refined iteratively through structured focus groups and then applied externally across institutions, providing preliminary evidence of transferability beyond the originating cancer center. Third, integration of qualitative and quantitative findings allowed us to evaluate not only domain-level maturity ratings, but also the organizational factors that may shape survivorship care implementation in practice.

This study also has important limitations. First, the CSMM was developed from the NCI survivorship standards but necessarily consolidated some standards into broader operational domains in order to create a usable maturity model. Although this approach was intended to improve interpretability and reflect how survivorship care is operationalized in practice, it may have reduced granularity for some survivorship care elements. Second, the internal focus groups were conducted within a single cancer center, and the external evaluation sample was modest. Notably, the external evaluators were drawn from NCI Survivorship Supplement funded institutions, centers that are among the most engaged in survivorship care nationally and would be expected to demonstrate relatively high organizational readiness to implement NCI survivorship guidelines. That maturity ratings nonetheless clustered at early stages in this group is a striking finding, suggesting persistent gaps in organizational readiness even in survivorship care focused settings. Consequently, these scores should not be interpreted as representative of broader oncology practice, as the external evaluation was intended to assess CSMM transferability rather than estimate national readiness.

Third, prioritization of patient needs was based on clinician perceptions rather than direct patient input. As a result, the patient-priority rankings should be interpreted as clinician-perceived patient priorities rather than a direct assessment of survivor perspectives. Finally, although the qualitative component yielded meaningful implementation themes, future work could strengthen the model through broader stakeholder engagement, including survivors, administrators, and operational leaders. Future research should evaluate the CSMM across a broader range of oncology practice settings and examine its utility for benchmarking survivorship readiness over time, guiding implementation strategies, and informing survivorship care quality improvement efforts. Inclusion of patient and caregiver perspectives will also be important for ensuring that future iterations of the model reflect survivor priorities more directly. As survivorship care standards continue to evolve, tools that help organizations assess and operationalize readiness will be increasingly important for translating national guidance into routine clinical practice.

## Conclusion

As survivorship care standards become more clearly defined, a central challenge for cancer centers and healthcare organizations is no longer whether survivorship care is important, but whether institutions are prepared to deliver it consistently and comprehensively. The CSMM offers a practical framework for assessing organizational readiness across key survivorship domains and for identifying where survivorship care infrastructure remains underdeveloped. By helping institutions distinguish areas of relative strength from areas requiring greater investment, the CSMM may support more strategic implementation planning, targeted resource allocation, and broader advancement of survivorship care delivery.

## Supplementary Material

Supplementary Files

This is a list of supplementary files associated with this preprint. Click to download.

- IMMSupplementalTables.docx

## Figures and Tables

**Table 1:** Participant Characteristics by Group. Values are presented as n (%).

Characteristic	BCM (n = 14)	NCISS (n = 12)	Overall (N = 26)
Age (overall)			
<30	1 (7.1)	0 (0.0)	1 (3.8)
30–39	4 (28.6)	2 (16.7)	6 (23.1)
40–49	3 (21.4)	5 (41.7)	8 (30.8)
50–59	4 (28.6)	3 (25.0)	7 (26.9)
60–69	1 (7.1)	2 (16.7)	3 (11.5)
70+	1 (7.1)	1 (8.3)	2 (7.7)
Gender			
Female	7 (50.0)	12 (100.0)	19 (73.1)
Male	7 (50.0)	0 (0.0)	7 (26.9)
Race			
White	9 (64.3)	11 (91.7)	20 (76.9)
Asian	4 (28.6)	0 (0.0)	4 (15.4)
Black or African American	0 (0.0)	1 (8.3)	1 (3.8)
Other	1 (7.1)	0 (0.0)	1 (3.8)
Ethnicity			
Not Hispanic	13 (92.9)	11 (91.7)	24 (92.3)
Hispanic	1 (7.1)	1 (8.3)	2 (7.7)
Years of Clinical Experience			
<5 years	2 (14.3)	1 (8.3)	3 (11.5)
5–10 years	3 (21.4)	2 (16.7)	5 (19.2)
11–15 years	3 (21.4)	2 (16.7)	5 (19.2)
16–20 years	4 (28.6)	2 (16.7)	6 (23.1)
>20 years	2 (14.3)	5 (41.7)	7 (26.9)
Education			
Doctoral	13 (92.9)	12 (100.0)	25 (96.2)
Masters	1 (7.1)	0 (0.0)	1 (3.8)
Occupation			
Oncologist	11 (78.6)	8 (66.7)	19 (73.1)
NP(Oncology)	1 (7.1)	0 (0.0)	1 (3.8)
Primary Care Provider	1 (7.1)	0 (0.0)	1 (3.8)
Psychologist	1 (7.1)	0 (0.0)	1 (3.8)
Palliative Care	1 (7.1)	0 (0.0)	1 (3.8)
Researcher	0 (0.0)	3 (25.0)	3 (11.5)

**Table 2:** Focus Group CSMM Assessment. Columns represent the individual maturity levels reported by BCM clinicians participating in cancer survivorship focus groups followed by the average for each cancer survivorship category amongst all BCM respondents and all NCISS awardee respondents in the last column.

Focus Group CSMM Assessment	C1	C2	C3	C4	C5	C6	C7	C8	C9	C10	C11	C12	C13	C14	BCM Avg
Specialty Care	1	1	1	1	2	3	2	4	2	2	1	2	4	3	2.07
Risk of Recurrence	2	2	3	2	2	2	2	4	2	2	1	2	2	2	2.14
Survivorship Care Planning	1	1	1	4	1	1	1	2	2	2	1	2	2	3	1.71
Physical & Psychological Effects	1	1	1	4	3	3	2	2	3	2	1	2	2	2	2.07
Lifestyle Behaviors & Supportive Services	1	1	1	3	2	2	1	1	1	1	1	2	2	2	1.50
Financial Hardship/Toxicity	1	2	2	2	1	3	1	2	1	3	1	2	2	2	1.79
Practical & Social Impacts	1	1	2	2	2	2	1	2	2	2	1	2	1	2	1.64

**Table 3.** Mean and Standard Deviation Statistical Summary Table. Maturity levels reported by BCM focus group participants taking the CSMM model assessment.

Category	Institution	Mean	SD	N
Specialty Care	BCM	2.071	1.072	14
	NCISS	1.917	0.793	12
	Overall	2	0.938	26
Risk of Recurrence	BCM	2.143	0.663	14
	NCISS	2.083	0.793	12
	Overall	2.115	0.711	26
Survivorship Care Planning	BCM	1.714	0.914	14
	NCISS	1.75	0.754	12
	Overall	1.731	0.827	26
Physical & Psychological Effects	BCM	2.071	0.917	14
	NCISS	1.5	0.522	12
	Overall	1.808	0.801	26
Lifestyle & Behavior Supportive Services	BCM	1.5	0.65	14
	NCISS	1.75	0.866	12
	Overall	1.615	0.752	26
Financial Hardship/Toxicity	BCM	1.786	0.699	14
	NCISS	2	0.603	12
	Overall	1.885	0.653	26
Practical & Social Impacts	BCM	1.643	0.497	14
	NCISS	2.5	0.674	12
	Overall	2.038	0.72	26

**Table 4:** Ordinal Logistic Regressions (BCM vs. NCISS). Ordinal logistic regression results comparing the BCM vs. NCISS group model assessments by cancer survivorship category. Categories are ordered by p-value. Only one category, Practical & Social Impacts, differs significantly between groups, indicating model transferability.

Category	Total	BCM N	NCISS N	OR NCISS vs BCM	95% CI	p-value
Practical & Social Impacts	26	14	12	24.58	2.46–246.05	0.0064
Physical & Psychological Effects	26	14	12	0.26	0.06–1.24	0.0917
Financial Hardship/Toxicity	26	14	12	2	0.43–9.34	0.3781
Lifestyle Behaviors & Supportive Services	26	14	12	1.71	0.38–7.64	0.4829
Survivorship Care Planning	26	14	12	1.29	0.30–5.53	0.7323
Risk of Recurrence	26	14	12	0.7424	0.1313–4.1983	0.7362
Specialty Care	26	14	12	0.8701	0.2125–3.5629	0.8465

**Table 5:** Qualitative Themes Identified from Focus Group Discussions. Institutional readiness barrier themes identified through qualitative thematic analysis of focus group discussions reflecting perceived barriers to implementation of cancer survivorship care.

**FG1**	**Reflections on Barriers to Cancer Survivorship Care Readiness**	**Themes**
	**Survivorship care is specific to each cancer diagnosis.** **Survivorship care is more resource intensive than the group had initially thought.** **“Lack of workflow clarity, about the process and roles”** **“Discontinuity of EHR systems across clinical practices”**	**Variation** **Time** **Process** **Technical**
FG2	“Survivorship care needs varies across cancer type and clinical settings. It is difficult for an institution to provide a one-size fits all solution to support it.” “Survivorship care is unfunded which translates into having no time reserved for these activities with the patient. Manpower is needed to successfully implement cancer survivorship guidelines.” “Clinician is bombarded with paperwork; screening information should be direct to patient, like a QR code in the lobby.”	Variation Cost & Time Process
FG3	“Care planning is multi-disciplinary, personalized and complex. Time is a limiting factor.” “A proactive survivorship checklist would be helpful. I like the idea of screening tools but do not box the physician in so that they have no flexibility or send too many EHR alerts causing alert fatigue. “Physical effects of cancer, follow-ups and referrals all need to be addressed by the physician, but the remaining survivorship axes could be handled by a case manager.” “More automated screenings are needed for survivorship care, but the institution all needs resources to act upon the data.”	Variation & Time Technical Process Resource
FG4	“The financial category is one of the ones that the patients are most concerned with and i’s also the only one (category) tha’s at a level one for both of us (oncologist raters). And the reason why it doesn’ get done at a higher level is because it requires having specialty people who understand the resources and can counsel them.” I think primary care should be handling some of the (cancer survivorship care) categories. I don’ know if I'm necessarily the person who's supposed to be doing it all.	Resource Process
FG5	“Tha’s always my concern with a lot of these guidelines, that they say, “Oh, you need to do this, that, and the other,” and the institution doesn’ have the people or the resources to actually follow through.” “Survivorship care has to be personalized to the patient. And i’s complex, but i’s very important. It should be multidisciplinary. It needs to be a proactive plan rather than planning as you go. But we don’ want to box the physician in, and we don’ want to send them a bunch of alerts that they must comply with if i’s not appropriate. And then it would be nice if there were some kind of preference list, but that could be done by a case manager or an intake nurse. There's a time limitation with the oncologist, so symptoms and side effects, referrals, and follow-ups should be handled by the oncologist, but the rest could be handled by the case manager.”	Time & Resource Process

## Data Availability

This is a study protocol; no data are currently available. Data generated during the study will be made available upon request after study completion.

## Availability of Analytic Code:

This is a study protocol. No analytic code are currently available.

## Availability of Materials:

There are no additional materials available for this study.
